# Strategy for Fabricating Multiple-Shape Memory Polymeric Materials Based on Solid State Mixing

**DOI:** 10.1021/acsmacrolett.4c00601

**Published:** 2025-01-12

**Authors:** Salim-Ramy Merouani, Roman Kulagin, Vladislav Bondarenko, Ramin Hosseinnezhad, Fahmi Zaïri, Iurii Vozniak

**Affiliations:** † The Bio-Med-Chem Doctoral School of the University of Lodz and Lodz Institutes of the Polish Academy of Sciences, Banacha 12/16, Lodz 90-237, Poland; ‡ Centre of Molecular and Macromolecular Studies, Polish Academy of Sciences, Sienkiewicza str., 112, Lodz 90363, Poland; § Institute of Nanotechnology, Karlsruhe Institute of Technology, Hermann-von-Helmholtz-Platz 1, 76344 Eggenstein-Leopoldshafen, Germany; ∥ Kryvyi Rih State Pedagogical University, Gagarin av. 54, 50086 Kryvyi Rih, Ukraine; ⊥ Laboratoire de Génie Civil et géo-Environnement, Université de Lille, IMT Nord Europe, JUNIA, Université d’Artois, ULR 4515-LGCgE, Lille 59000, France

## Abstract

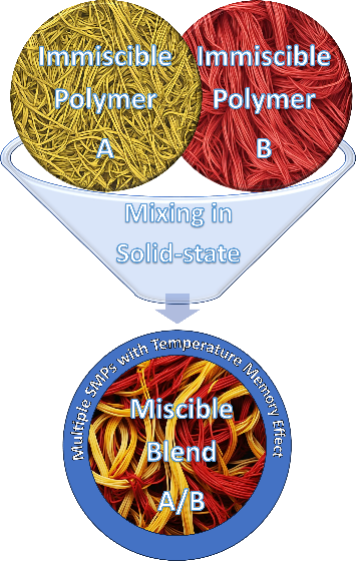

Traditionally, multiple shape memory polymers (multiple-SMPs) are created by forming either immiscible blends with high phase continuity (cocontinuous or multilayer phase morphology) or miscible blends that exhibit compositional heterogeneity at the nanoscale. Here, a new strategy for the fabrication of multiple-SMPs is proposed. It consists of the possibility of homogeneous mixing of immiscible polymers in the solid state under high pressure and shear deformation conditions. The blends formed in this way exhibit homogeneity of mixing down to the nanoscale, up to 40–95 nm. The transition from immiscible to miscible blends leads to an improvement not only in shape memory but also in the mechanical performance of the blends formed. Polypropylene (PP) and polystyrene (PS) were selected as pairs of immiscible polymers. The method of solid phase mixing is high pressure torsion (HPT). It was shown that the HPT-processed 50% PP/50% PS blend is able to exhibit an excellent triple shape memory effect (shape fixation of ∼94–95%, and recovery of ∼85–95%) with widely tunable (low and high) transition temperatures.

Shape memory polymers (SMPs) are smart materials that are able to fix the temporary programmed shape and restore the permanent shape under external stimuli.^1−3^ Of late, multiple-shape memory polymers (multiple-SMPs) have been developed which can store two or more temporary shapes and return to the permanent shape sequentially.^4−8^ In general, there are two main strategies to obtain multiple-SMPs

One strategy for the development of multiple-SMPs is based on the inclusion of several separate transitions into one material. Typical examples are macroscopically homogeneous polymers (graft and block copolymers, interpenetrating polymer networks, liquid-crystal elastomers, immiscible blends) consisting of microseparated phases, polymer multilayers and composites.^9−15^ As a rule, the maximum number of shapes that can be memorized correlates with the number of individual transitions. In this case, the necessary condition for creating optimal multiple-SMPs is the creation of a cocontinuous phase morphology with high phase continuity^16−18^ or a multilayer structure assembled by the parallel distributed layers (the latter has the highest phase continuity at any component ratio).^19−24^ The high phase continuity and numerous interfaces between the layers and phases contribute to improving the miscibility and overall SMP performance (balance between shape fixation and shape recovery).

The other strategy is to mix miscible polymers to obtain a single, broad thermal phase transition such as the broad glass or melt transition.^25−28^ The condition is that the homogeneity of mixing of miscible polymers should not be achieved at the molecular level but at the nanoscale. Such compositional heterogeneity at the nanoscale enables the formation of different *T*_g_ nanodomains with different amounts of mixed polymer chains that can be selectively activated during programming.^24,28^ In this way, the single broad thermal transition is considered as a collection of an infinite number of discrete thermal transitions corresponding to infinitely sharp transition temperatures that are continuously distributed across the broad transition.^28−32^ In a broad thermal phase transition range, different transition temperatures can be freely chosen, and accordingly, several temporary shapes have been achieved. Based on this principle, the temporary shapes of a multiple-SMP can be fine-tuned without the need to introduce further compositions, which is a real advantage for the robustness, recyclability, and reuse of multiple-SMPs. Even a single phase, as in the case of Nafion, a cross-linked fluoropolymer, which has a broad glass transition, is sufficient to exhibit at least quadruple-shape memory effect.^29,30^ The temporary shapes can therefore be adjusted by varying the applied mechanical energy at each deformation temperature and the recovery of each temporary shape can be facilitated near the corresponding deformation temperature, which is known as the temperature memory effect.^33,34^ The main efforts are focused on extending the thermal transition range, mainly through the conventional melt mixing technique.^35−39^

The first strategy has largely prevailed, as it is based on the use of immiscible polymers, of which the number is enormous. However, since the morphology of the immiscible blend is easily influenced by the viscosity ratio of the components, the interfacial tensions or the processing parameters,^40−43^ especially for the multiphase system, tailoring the structure of a multiphase blend to produce a cocontinuous multiple SMP using the melt processing methods, while possible, is sophisticated. There are also certain limitations with the multiple-SMP systems obtained by synthesis and other complex molecular designs.^2,3^ The second strategy is less popular, as it is based on the use of polymers with a wide thermally reversible phase transition or miscible blends, the number of which is limited. However, its advantage is the impressive principle based on the ability of polymers to memorize programmed temperatures, thus providing a flexible approach to adjust shape memory behavior without changing the chemical composition of the polymers.

In the work reported here, a new approach is introduced to achieve homogeneous mixing of immiscible polymers on the nanoscale and the formation of a broad glass transition in the formed blends. This is achieved by shear deformation under a high pressure in the solid state. The difference between mixing in the solid state and mixing in the melt is that in the former, the processes of decaying and subsequent merging and coarsening of the polymer phases, which cause phase separation of the immiscible polymer phases, do not take place.^44^ Using the example of the polypropylene–polystyrene (PP/PS) system, a solid-state modified blend is obtained that is characterized by a broad glass transition and enables the formation of at least triple-SMPs. High pressure torsion (HPT), which is often used for the formation of immiscible metal alloys, is chosen as the method for realizing high pressure and shear deformation in the solid phase.^45−47^

Figure 1 panels A and B show the results of DSC and TGA studies indicating the degree of mixing of the polymer phases at the micro level in the initial and HPT-processed PP/PS blends. For the initial PP/PS blend, which is an immiscible blend and exhibits heterogeneity of mixing at the micro level, a well-defined glass transition step for polystyrene is observed on the DSC curve, and a two-step reaction corresponding to separate thermal degradation of polystyrene and polypropylene is observed on the TGA curve. At the same time, in the HPT-processed PP/PS blend, no glass transition step for polystyrene is seen and the thermal degradation of polystyrene and polypropylene occurs simultaneously, as evidenced by the registration of a one-step reaction on the TGA curve. This indicates the homogeneity of the mixing of the studied polymers at the microscale. The SEM results in Figure 1S further confirm the homogeneous mixing of the polymer phases at the microscale for the HPT-processed PP/PS blend. Moreover, the results of the solid-state NMR studies in Figure 1C,D show that in the case of the HPT-processed PP/PS blend homogeneous mixing of the polymer phases takes place down to the nanoscale. In contrast to the initial PP/PS blend, which is characterized by significantly different relaxation times (^1^H spin–lattice relaxation time in the laboratory frame, *T*_1_) for the PP and PS components, the HPT-processed PP/PS blend exhibits relaxation times very close to each other, indicating that the mixing of the polymers takes place in the range of a few tens of nanometers.^48^ It is estimated that PP and PS in the HPT-processed blend are intimately mixed on a scale of 40–95 nm. The same conclusion follows from the analysis of the ^1^H spin–lattice relaxation times in the rotating frame, *T*_1r_, which characterize the scale at which homogeneity is observed. It is evident that the difference between the *T*_1r_ values for PP and PS is reduced from 17.29 to 6.32 ms by the HPT treatment and is thus characteristic of miscible blends.^48,49^

**Figure 1:**
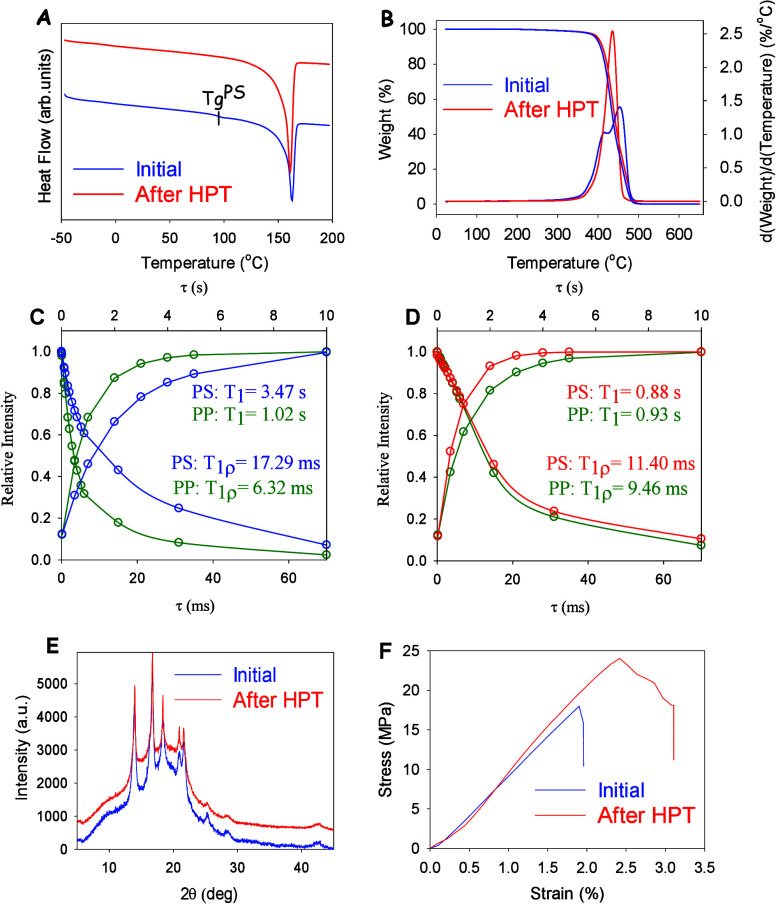
(A) DSC and (B) TGA thermograms of initial (nonprocessed) and HPT-processed blends. *T*_1_ recovery and *T*_1ρ_ decay relaxation time of initial (C) and HPT processed blend (D) measured by ^13^C CP/MAS NMR. (E) WAXS and (F) stress–strain curves under uniaxial tensile test.

It is important to note that the corresponding amorphous phases of the polymers to be blended are subject to mixing. In addition, the proportion of the crystalline phase of polypropylene remains practically unchanged according to the WAXS data (Figure 1E). The latter is particularly important for maintaining the proportion of the so-called permanent domains, which are responsible for high values of the shape fixation ratio. It can also be assumed that the transition from immiscible to miscible blends should significantly increase the proportion of the interphase, which improves interphase adhesion. The interphase is considered as so-called permanent domains^22^ and also contributes to the formation of the broad glass transition.^20^

Figure 2A,B shows a typical stress–strain-temperature curve as a function of time for initial and HPT-processed PP/PS blends. As can be seen, the transition temperatures were set at 30 and 45 °C, with an overall stretching to 40% strain. As summarized in Table 1, the shape fixity ratio, *R*_f_, quantifies the ability to fix the switching domains and is influenced by the interaction between the polymer chains during the deformation process, while the shape recovery ratio, *R*_r_, quantifies the extent to which the polymer memorizes its original/temporary shape, as it takes into account the degree of strain that is recovered.

**Figure 2:**
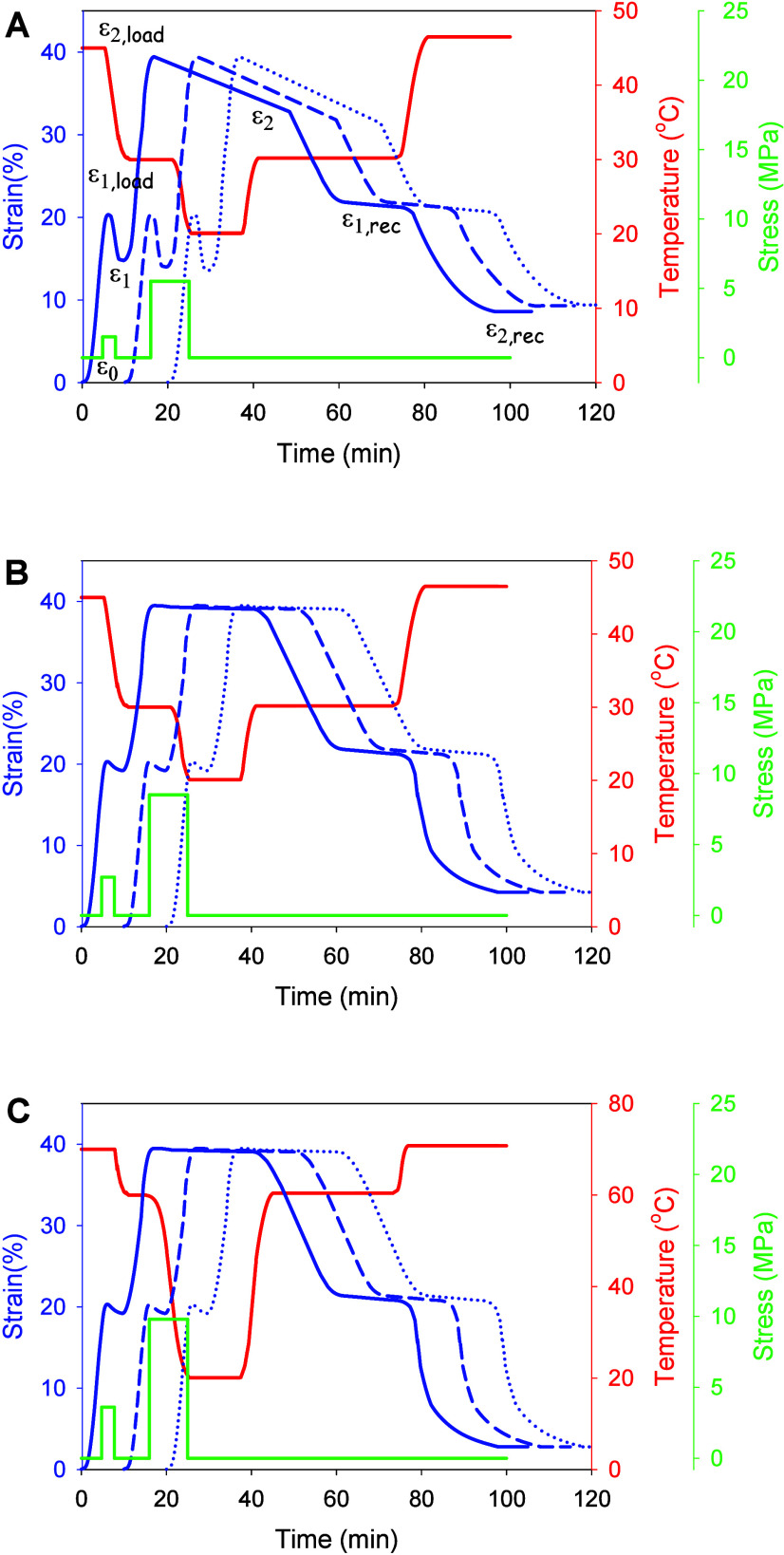
Stress–strain–temperature curves as a function of time for (A) initial and (B,C) HPT-processed PP/PS blends. Strain curves are shifted along the time axis (10 and 20 min increments for second and third cycles, respectively) for clarity of presentation. The transition temperatures are 30 and 45 °C (A and B) and 60 and 70 °C (C).

**Table 1:** Shape Memory Capabilities of the Initial and HPT-Processed PP/PS Blends

		Shape memory capabilities			
Blend	Cycle number^a^	*R*_f1_, %	*R*_r1_, %	*R*_f2_, %	*R*_r2_, %
initial	1a	71	65	74	62
	2a	68	60	70	59
	3a	66	58	68	57
HPT-processed	1a	95	87	95	85
	2a	94	87	95	84
	3a	94	86	95	85
	1b	96	95	95	92
	2b	96	95	95	92
	3b	96	95	95	91

a a - SMP cycling, with programming at 30 and 45 °C. b - SMP cycling, with programming at 60 and 70 °C.

As expected, the initial PP/PS blend exhibits a relatively low *R*_f_ value of 71% and an *R*_r_ value of 65% for the first temporary shape and an *R*_f_ value of 74% and an *R*_r_ value of 62% for the second temporary shape. In contrast, the HPT-processed PP/PS blend has a much better shape memory with an R_f_ value of 95% and an *R*_r_ value of 87% for the first temporary shape and an R_f_ value of 95% and an *R*_r_ value of 85% for the second temporary shape. It is known that the recovery is primarily driven by the entropy due to the elasticity of the polymer chains, the so-called entropic elasticity.^50^ Based on entropic considerations, the random arrangement of the chains possesses much higher entropy than the chains held in the temporary state.^51^ The improvement in recovery is therefore largely attributed to the increase in conformations caused by the homogeneous mixing of the polymer phases at the nanoscale, which gives the HPT-processed PP/PS blend an advantage in the entropy-driven recovery process.^52^ The permanent domains that hold the frozen amorphous chains are attributed to the physical interaction and entanglement between amorphous PP and PS segments as well as to the crystalline regions of PP. They contribute significantly to the high shape fixity and recovery. Since the degree of crystallinity of PP remained practically unchanged by the HPT treatment, it can be assumed that the key role in improving the shape fixity and shape recovery ratio was played by a significant increase in the proportion of interphase, which is much higher in miscible blends compared to immiscible blends.^53^ The remarkable improvement in shape memory performance in the case of the HPT-processed PP/PS blend can also be attributed to the more effective load transfer between PS and PP switching domains due to the homogeneous mixing of the two polymers. Furthermore, strain–time curves demonstrate that immiscible blends exhibit more pronounced creep behavior (i.e., an increase in strain over time under constant stress) compared with miscible blends (Figure 2). Creep, as a form of viscous flow, compromises shape fixity ratios and dissipates part of the stored elastic energy in the SMPs, leading to lower recovery ratios and reduced performance during cycling.

The stability of the shape memory behavior of the PP/PS blends was then tested for up to three cycles. Figure 2 shows the effects of the cycles on the shape memory behavior. It can be seen that *R*_f_ and *R*_r_ remain practically unchanged for the HPT-processed PP/PS blend, whereas they deteriorate for the initial blend as the number of cycles increases (Table 1). The latter could be related to a weak interface in the case of the immiscible blend and a possible enhancement of phase segregation during cycling.^16,28^

The DMTA temperature sweep data in Figure 2S show that for the nonprocessed PP/PS blend, three loss modulus peaks are observed with maxima at temperatures of 15, 65, and 120 °C. The peaks at 15 and 120 °C are associated with the β-transition of PP and the α-transition of PS, respectively. In the temperature range of 50–60 °C, a plateau region is typically observed as a result of the α-transition of PP, attributed to the presence of a “rigid” amorphous phase in the crystals.^54,55^ At the same time, the presence of a small wide peak in this region indicates the formation of a minor fraction of interphase layers in the immiscible nonprocessed PP/PS blend. This is likely due to the 50 wt %/50 wt % component ratio, which leads to the formation of a cocontinuous morphology, maximizing the interaction between the components. In the case of the HPT-processed PP/PS blend, a single broad transition peak is observed, with maxima of the peaks at 15, 65, and 120 °C barely detectable. This is associated with the formation of a large number of interphase layers and the creation of a homogeneous amorphous phase in the miscible HPT-processed PP/PS blend. The storage modulus and tan δ data correlate with the loss modulus results, showing more uniform behavior in the HPT-processed blend, indicating greater homogeneity and interphase formation (Figure 2SB,C).

A broad glass transition makes it possible to choose any transition temperature that lies within this transition. It is necessary to ensure that the processes of programming and recovering temporary shapes do not overlap. To this end, the quenching temperature at which the high temperature temporary shape is fixed must be higher than the programmed temperature of the low temperature temporary shape. Otherwise, the programming of one temporary shape would be accompanied by a partial recovery of the other temporary shape of the multiple SMPs. For the initial PP/PS blend, the temperature range in which full free strain recovery occurs under continuous heating conditions at 2 °C/min was 15 °C, whereas this temperature range was reduced to 8 °C for the HPT-processed PP/PS blend (Figure 3S). This is probably due to the formation of a large number of interphase layers that occur during the homogeneous mixing of the polymers at the nanoscale and represent a network of physical entanglements. The possibility of tuning the transition temperature is shown in Figure 2C. The transition temperatures were set to 60 and 70 °C, and the overall stretching was 40% strain. It can be seen that *R*_f_ of 96% and an *R*_r_ of 95% are achieved for the first temporary shape and *R*_f_ of 95% and *R*_r_ of 92% for the second temporary shape.

According to existing concepts,^53,56^ broad glass transitions of miscible blends could be considered as a collection of individual glass transitions that could act as an individual shape memory element. In this case, the switching domains with a specific *T*_g_ consist of different amounts of oriented PP and PS chains that can be selectively activated during the programming step. In this respect, only the independent memory components with a transition temperature below the programming temperature are capable of movement during the programming step.^34^ Therefore, the higher values of *R*_f_ and *R*_r_, observed when programming at higher temperatures, are probably due to the fact that, at such temperatures, a larger proportion of PS chains are involved in elastic energy storage for shape recovery. In addition, a higher degree of mobility of the less frozen PS chains, which subsequently produce less irreversible deformation when the deformation stress is released, results in a higher *R*_f_ value. Figure 4S presents digital images of the original, temporary, and permanent shapes of nonprocessed and HPT-processed PP/PS blends.

Finally, the HPT-processed PP/PS blend exhibits a greater maximum recovery stress σ_r_ and has a higher modulus of elasticity *Ε* and yield strength σ_*y*_ compared to those of the initial blend (Figure 1F, Table 2). The significantly higher values of σ_r_, *Ε*, and σ_*y*_ show that the immiscible–miscible blend transition ends up multiple-SMPs with better mechanical performance. In particular, the maximum recovery stress increases from 3.7 to 6.1 MPa, the modulus of elasticity from 947 to 1757 MPa and the yield strength from 20.0 to 24.5 MPa.

**Table 2:** Mechanical Properties of the Initial and HPT-Processed PP/PS Blends

Blend	σ_r_, MPa	*Ε*, MPa	σ_*y*_, MPa
initial	3.7 ± 0.2	947 ± 76	20.0 ± 0.6
HPT-processed	6.1 ± 0.3	1757 ± 140	24.5 ± 1.2

In summary, a new approach was proposed for the development of multiple-SMPs based on the homogeneous mixing of immiscible polymers in the solid state under high pressure and shear deformation. The mixing of immiscible polymers in the solid phase enables the production of homogeneous polymer blends on the nanoscale and the formation of a broad glass transition in the blends formed. This enables the selection of a wide range of possible transition temperatures while maintaining high *R*_f_ and *R*_r_ values even during the cycle. The latter is due to the formation of a large number of interphase boundaries, which is an essential feature of the transition from immiscible to miscible blends. Not only the shape memory but also the mechanical properties of blends processed in the solid state are improved. This possibility is demonstrated using the example of a blend of immiscible polymers, PP and PS. HPT is a scheme that enables blending of polymers in the solid phase. The HPT-processed PP/PS blend allows the formation of at least triple shape memory effect and a smooth change in transition temperatures. The proposed approach is universal and allows blending of different pairs of immiscible polymers. This creates a wide possibility to regulate the transition temperatures, i.e., to obtain both low temperature triggered and high temperature triggered SMPs. The former with transition temperatures close to body temperature are particularly promising for advanced medical applications; the latter with high transition temperatures have a broad application potential in harsh environments. The prospects of solid-state mixing are also that an immiscible ternary or multicomponent polymer system can be converted into miscible blends and thus be amenable to the formation of multiple SMPs. In contrast, the compatibilization of such systems would be associated with the difficulty of producing an effective complex multiphase compatibilizer, as this is mainly used to compatibilize binary blends.

## Supporting Information Available

Experimental details and characterization data (PDF)

## Abbreviations

HPT: high pressure torsion
multiple-SMPs: multiple shape memory polymers
PP: polypropylene
PS: polystyrene.
