# Correction to “Strategy for Fabricating Multiple-Shape Memory Polymeric Materials Based on Solid State Mixing”

**DOI:** 10.1021/acsmacrolett.5c00158

**Published:** 2025-04-07

**Authors:** Salim-Ramy Merouani, Roman Kulagin, Vladislav Bondarenko, Ramin Hosseinnezhad, Fahmi Zaïri, Iurii Vozniak

The following author emails should have been included in the original paper:

Salim-Ramy Merouani: salim.merouani@edu.uni.lodz.pl.

Ramin Hosseinnezhad: ramin.hosseinnezhad@cbmm.lodz.pl.

Roman Kulagin: roman.kulagin@kit.edu.

Fahmi Zaïri: fahmi.zairi@polytech-lille.fr.

In the original paper, authors Ramin Hosseinnezhad and Iurii Vozniak had the following affiliation: The Bio-Med-Chem Doctoral School of the University of Lodz and Lodz Institutes of the Polish Academy of Sciences, Lodz 90-237, Poland. That affiliation is corrected to the following: Centre of Molecular and Macromolecular Studies, Polish Academy of Sciences, Lodz 90363, Poland

