# Supplementary material for: Strategy for Fabricating Multiple-Shape Memory Polymeric Materials Based on Solid State Mixing
Source: ACS Macro Lett. 2025 Jan 12;14(2):129–34. doi: 10.1021/acsmacrolett.4c00601 (PMC11841025; doi:10.1021/acsmacrolett.4c00601)
Supplement: Supplementary file 1 — mz4c00601_si_001.pdf [file mz4c00601_si_001.pdf]

Supporting Information for Publication

Strategy for fabricating multiple-shape memory polymeric materials based on solid state mixing

Salim Merouani[1,2], Roman Kulagin[3], Vladislav Bondarenko[4], Ramin Hosseinnezhad[1], Fahmi Zairi[5], Iurii Vozniak[1]*

[1] The Bio-Med-Chem Doctoral School of the University of Lodz and Lodz Institutes of the Polish Academy of Sciences, Banacha 12/16, Lodz, 90-237, Poland; salim.merouani@edu.uni.lodz.pl

[2] Centre of Molecular and Macromolecular Studies, Polish Academy of Sciences, Sienkiewicza str., 112, Lodz 90363, Poland; ramin.hosseinnezhad@cbmm.lodz.pl, iurii.vozniak@cbmm.lodz.pl

[3] Karlsruhe Institute of Technology, Institute of Nanotechnology, Hermann-von-Helmholtz-Platz 1, 76344 Eggenstein-Leopoldshafen, Germany; roman.kulagin@kit.edu

[4] Kryvyi Rih State Pedagogical University, Gagarin av. 54, 50086, Kryvyi Rih, Ukraine;

[5] Laboratoire de Génie Civil et géo-Environnement, Université de Lille, IMT Nord Europe, JUNIA, Université d'Artois, ULR 4515-LGCgE, Lille 59000, France; fahmi.zairi@polytech-lille.fr

Experimantal details and characterization data

Materials: polypropylene (PP; Moplen HP500N) with MFI = 12.0 g/10 min (230°C/2.16 kg, ASTM D1238), and polystyrene (PS; Synthos PS GP 585X) with MFI = 8.0 g/10 min (200°C/5.0 kg, ASTM D1238) were used in the experiments.

Sample Preparation: An initial blend with 50 wt.%/50 wt.% polymer components was prepared on the Flashforge Creator pro three-dimensional printer by alternately depositing layers of blended polymers. The angular displacement of the layers was 90^0. For printing two-layer blends, an additional technological layer was required to guide the nozzle out of the printing zone when changing the material. The temperatures of the extruder were as follows: 250 °C /250 °C. The temperature of the support was: 60 °C. In all cases, the thickness of the melt layers was 0.2 mm. Melt blends were prepared using a Brabender batch mixer (Duisburg, Germany) operating at 190 °C for 10 minutes at 100 rpm.

High Pressure Torsion Setup: The samples with an initial diameter of 15 mm and a thickness of 1.5 mm underwent deformation using a self-made HPT installation (W. Klement GmbH, Lang, Austria) with a quasi-constrained anvil design - one anvil featured a 0.3 mm groove, and the second was flat.

Schematic representation of high-pressure torsion (HPT).

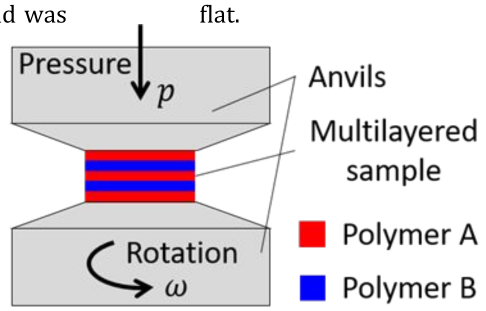

All experiments were carried out according to the following scheme. First, the sample was unloaded until the required pressure level (1GPa) was reached. Then, one of the anvils was brought into rotation with a given angular velocity, while the regulator automatically maintained the required pressure level throughout the whole deformation. All experiments were performed at ambient temperature, the number of revolutions was 50, the rotation speed was 5 rpm.

Characterization: Solid-state NMR spectra were recorded on a Bruker AVANCE III WB 400 MHz spectrometer. The spinning rate for all experiments was 6 kHz. 13C cross-polarization magic-angle spinning spectra were obtained under two-pulse phase modulating proton decoupling. A recycle delay was 2 s. 1H spin-lattice relaxation time measurements both in the laboratory and in rotating frames T_1 and $T_{1\rho}$ respectively were measured at room temperature by saturation recovery method and spin lock method, respectively. Thermal behavior of samples was probed with DSC Q20 differential scanning calorimeter (TA Instruments) during heating with the rate of 10 °C/min. Samples of the 7–8 mg mass were cut out from initial and HPT-processed blends and crimped in standard Al pans. The DSC cell was purged with dry nitrogen during the measurements (50 ml/min). The DSC curves shown in Figure 1A correspond to the second heating scan. The first heating scan was used to remove residual stresses and to erase any kinetic effects. The TGA was performed from room temperature to 600 °C at 10 °C/min with a Rigaku Instrument Thermo plus TG 8120 in a nitrogen atmosphere. The X-ray measurements were carried out with an Aeris XRD diffractometer (Malvern Panalytical) operating at 40 kV and 7.5 mA. The material before and after HPT were grinded for the XRD measurements to randomize the polymer crystal orientation. The mechanical properties of initial and HPT processed blends were evaluated using a tensile testing machine (Instron, Model 5582). The samples were cut into a rectangular shape measuring 15 × 5 mm, with an initial distance of 10 mm between the clamps. The crosshead speed was set at 5%/min, and the sample under each condition was tested five times. Yield strength and modulus of elasticity were calculated based on the stress–strain curve of each sample. Due to the small size of the HPT-treated specimens, it was not possible to evaluate the strain at break of the blends as the lack of an oar-shaped specimen resulted in stress concentration at the locations where the specimens were clamped in the grips, leading to their premature failure. Scanning electron microscopy (SEM) was conducted using a JEOL JSM-5500 LV scanning electron microscope (Tokyo, Japan). Prior to examining the morphology of cryogenically fractured samples, the PP/PS blends were permanganic-etched as reported by Galeski et al. [Galeski, A., Bartczak, Z., Vozniak, A., Pawlak, A., Walkenhorst, R., "Morphology and Plastic Yielding of Ultrahigh Molecular Weight Polyethylene," Macromolecules, 2020, 53(14), pp. 6063–6077].

Thermally-activated shape memory characterization of samples was conducted using Q800 DMA instrument with the film tension clamp under controlled strain and controlled force modes. The triple shape memory properties were enforced by primary strain deformation of 20% applied at 45 or 70 °C. The strain was then kept constant. The strain was then held constant while the sample was cooled. The second deformation was performed at 30 or 60 °C to increase the strain from 20 to 40 %. The strain was held constant until the sample had cooled to room temperature and then released. To observe the memory effect, the samples were heated to 32 or 61 °C for 30 minutes to recover the shape in the first step. Later, the temperature was increased to 47 or 72 °C to visualize the second memory shape. The recovery stress of PP/PS blends was performed under iso-strain conditions (strain=20 %) from 20 °C to the deformation temperature (shape memory programming) at 5 °C/min.

The shape fixity R_f and the shape recovery R_r ratios were calculated using the following equations: $R_{f1} = \frac{\varepsilon_1 - \varepsilon_0}{\varepsilon_{1,load} - \varepsilon}$; $R_{f2} = \frac{\varepsilon_2 - \varepsilon_1}{\varepsilon_{2,load} - \varepsilon}$; $R_{r1} = \frac{\varepsilon_{rec2} - \varepsilon_{rec1}}{\varepsilon_1 - \varepsilon_0}$; $R_{r2} = \frac{\varepsilon_2 - \varepsilon_{rec1}}{\varepsilon_2 - \varepsilon_1}$

ε_0 is the initial strain, $\varepsilon_{1,load}$, $\varepsilon_{2,load}$ are the deformed strains after loading; ε_1, ε_2 are the fixed strains after cooling and unloading; and ε_{rec1}, ε_{rec2} are the recovery strains. Subscriptions 1 or 2 indicate the values for the first and second temporary shapes. The corresponding characteristic strains are shown in Figure 2A.

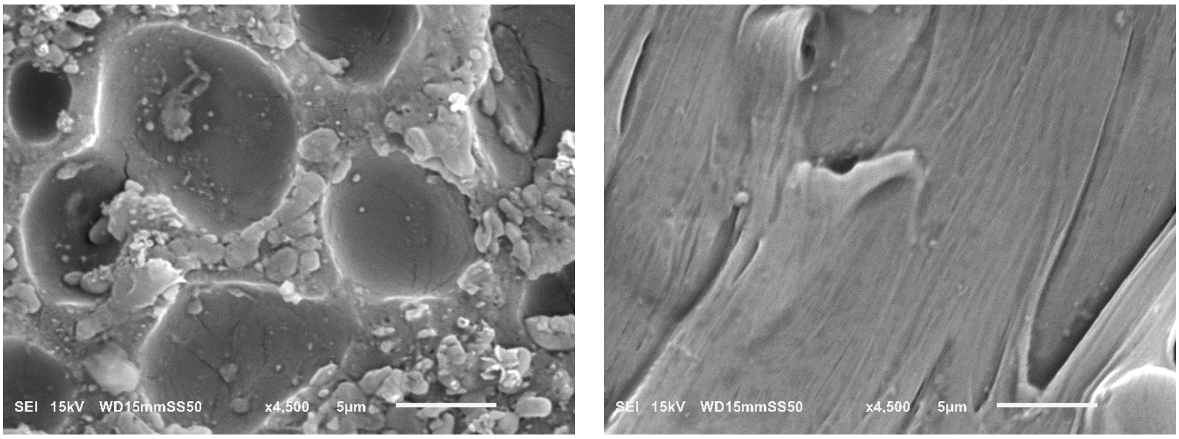

Figure 1 S. Scanning electron microscopy (SEM) images of PP/PS blends: (left) initial blend and (right) HPT-processed blend.

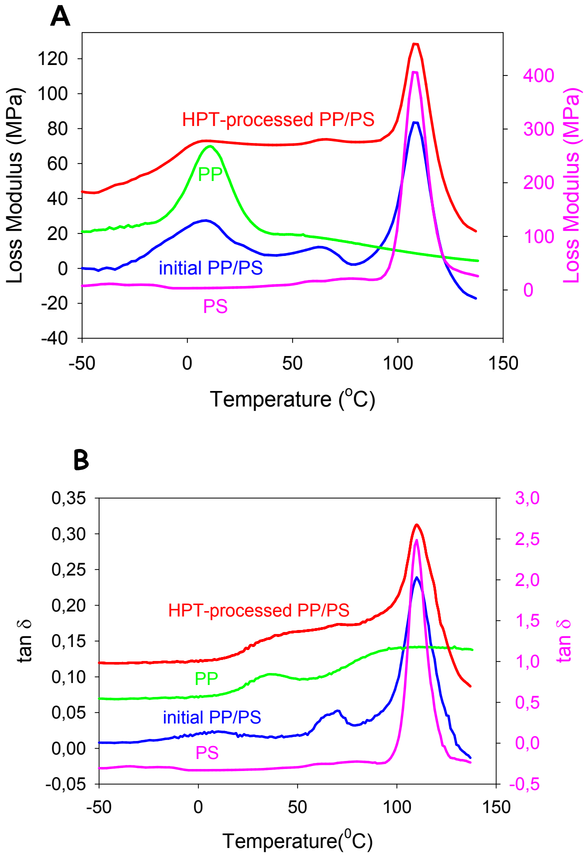

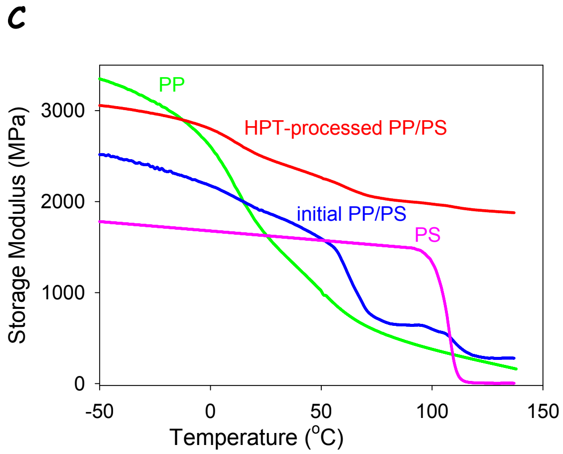

Figure 2 S. Loss modulus (A), tan δ (B), and storage modulus (C) as a function of temperature for PP, PS, and PP/PS blends, both unprocessed and HPT-processed. For clarity, the loss modulus curves for the PP/PS blends are offset by 20 MPa (upward for HPT-processed and downward for unprocessed), and the tan δ curves by 0.05 (upward for HPT-processed and downward for unprocessed).

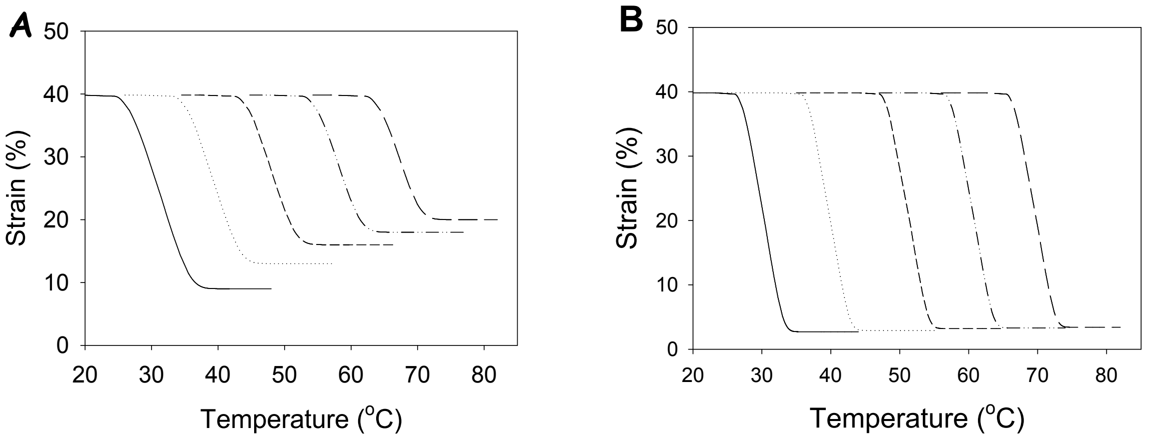

Figure 3 S. Free-strain recovery behavior observed upon heating at 2 °C/min for the (A) initial and (B) HPT-processed PP/PS blends, stretched at 30, 40, 50, 60, and 70 °C (curves are arranged from left to right).

A)

initial | Fixing | Recovery

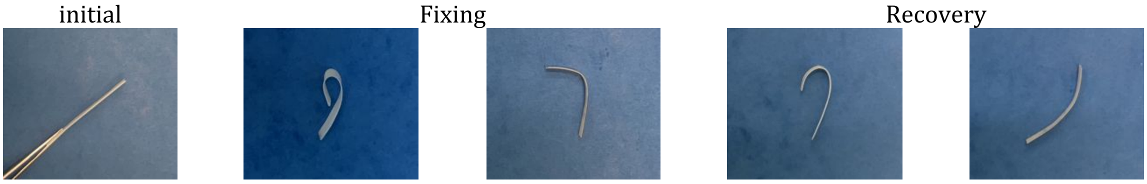

Shape 1 | Shape 1→2 | Shape 2→3 | Shape 3→2 | Shape 2→1

B)

HPT-processed | Fixing | Recovery

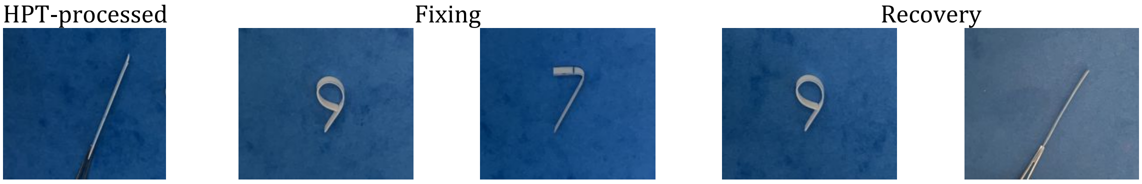

Shape 1 | Shape 1→2 | Shape 2→3 | Shape 3→2 | Shape 2→1

Figure 4 S. Illustration of the triple-shape memory behavior of initial and HPT-processed PP/PS blends. The transition temperatures are 60 and 70 °C.
